# Supplementary material for: The MeshCODE to scale—visualising synaptic binary information
Source: Front Cell Neurosci. 2022 Nov 18;16:1014629. doi: 10.3389/fncel.2022.1014629 (PMC9716431; doi:10.3389/fncel.2022.1014629)
Supplement: Supplementary file 6 [file Data_Sheet_1.docx]

# Supplementary information for

# The MeshCODE to scale – Visualising synaptic binary information.

Samuel F H Barnett^1^ and Benjamin T Goult^2*^.

^1^Max Planck Institute for Medical Research, Heidelberg, 69120, Germany.

^2^School of Biosciences, University of Kent, Canterbury, Kent, CT2 7NJ, UK.

# Supplementary Figures


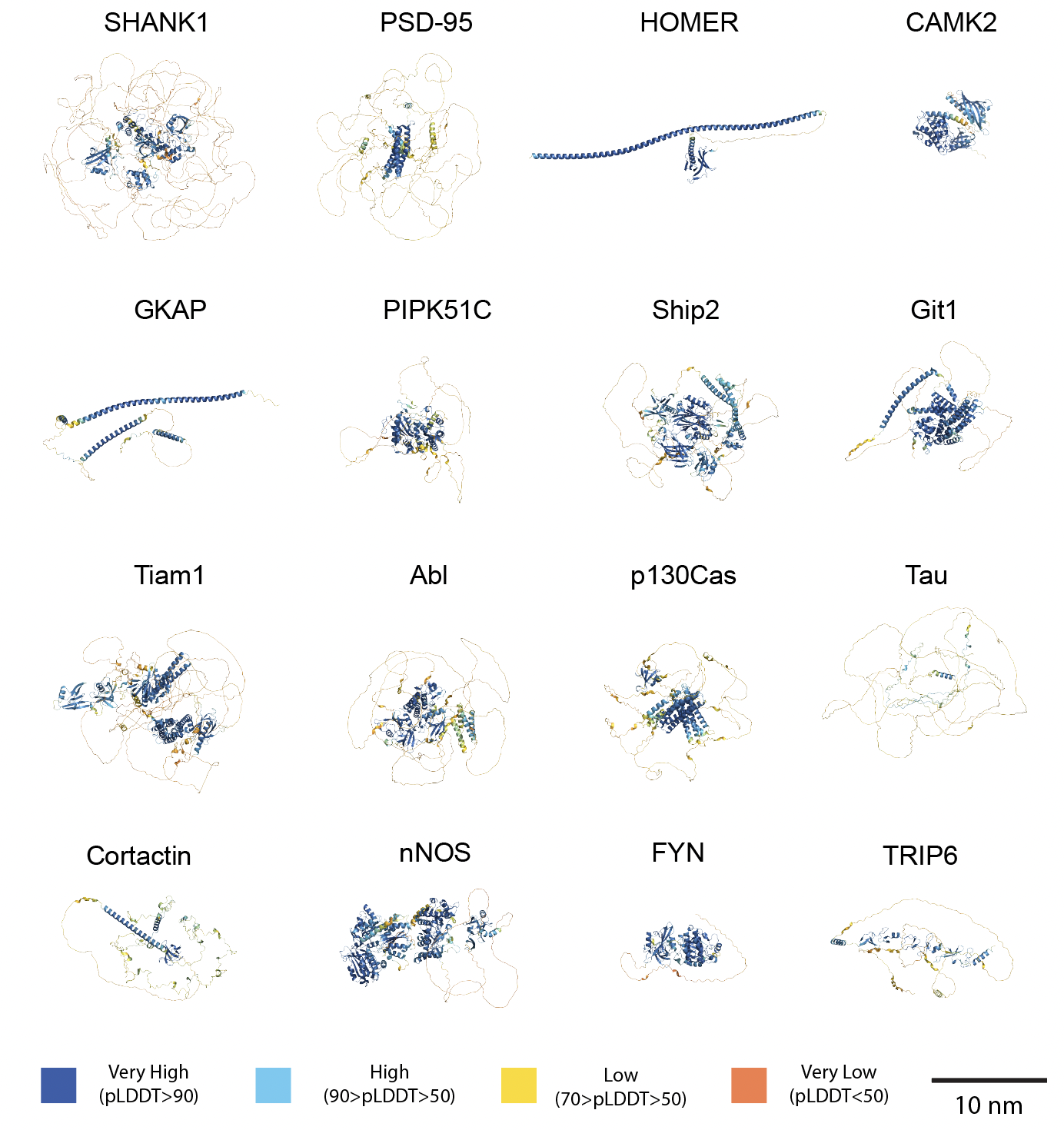


**Supplementary Figure 1: Selection of synaptic proteins and Integrin Adhesion Complex proteins with large expanses of unstructured regions generated using AlphaFold.** The colour coding (per Residue Confidence Score – pLDDT) indicates how often amino acids end up with the same neighbours. Dark blue indicates stable domains. Orange indicates unstructured regions.

**
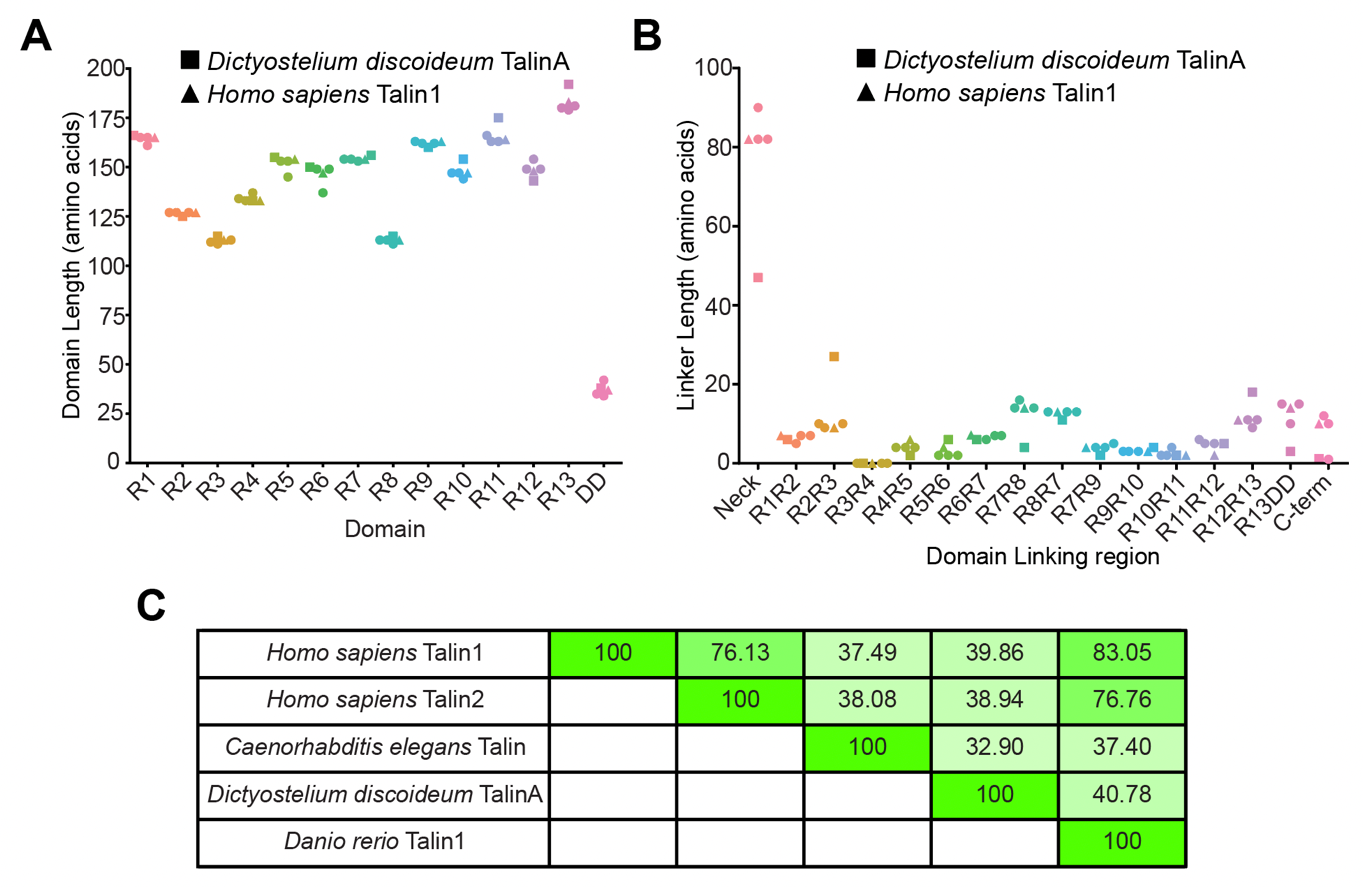
**

**Supplementary Figure 2: Talin protein domain structure is evolutionarily conserved. A)** Measurement of domain sizes of talin proteins from different species that have diverged over the last 0.6-1 billion years with *Homo sapiens* Talin1 and *Dictyostelium discoideum* TalinA shown to highlight this conservation. Other proteins are *Homo sapiens* Talin2, *Caenorhabditis elegans* Talin, and *Danio rerio* (zebrafish) Talin1. **B)** Measurement of the amino acid sequence that bridge the talin domains in A. e.g. R1R2 denotes the linker region between the R1 and R2 domains. C-term represents the amount of unstructured sequence following the dimerization domain. **C)** Similarity matrix showing the sequence conservation between the proteins in A-B.

# Supplementary Table

**Supplementary Table 1 – The UniProt accession codes of the proteins analysed in this study.**

Proteins visualised in Fig 5:

| Protein Name | UniProt Code |
| --- | --- |
| Talin | P26039 |
| Vinculin | P18206 |
| FAK | Q05397 |
| DLC1 | Q96QB1 |
| EGFR | P00533 |
| KANK | Q14678 |
| Paxillin | P49023 |
| RIAM | Q7Z5R6 |
| Tensin2 | Q63HR2 |
| ATAT1 | Q5SQI0 |
| CDK1 | P06493 |

Proteins visualised in Supplementary Figure 1:

| Protein Name | UniProt Code |
| --- | --- |
| SHANK1 | Q9Y566 |
| PSD-95 | Q9P1A6 |
| HOMER | Q86YM7 |
| CAMK2 | Q9UQM7 |
| GKAP1 | Q5VSY0 |
| PIP5K1C | O60331 |
| SHIP2 | O15357 |
| GIT1 | Q9Y2X7 |
| TIAM1 | Q13009 |
| ABL1 | P00519 |
| P130CAS | P56945 |
| TAU | P10636 |
| CORTACTIN | Q14247 |
| nNOS | P29475 |
| FYN | P06241 |
| TRIP6 | Q15654 |

# Supplementary Movies

**Supplementary Movie 1. Talin R3 as the paradigm of a force-dependent binary switch.** All 13 talin switches are shown in the folded, 0 state. Mechanical force opens the R3 switch, causing it to switch binding partners between RIAM (yellow) bound to R3(0) and two vinculin (purple) bound to the R3(1) states. <https://youtu.be/1R1CO9cOoRE>

**Supplementary Movie 2. Talin undergoes dramatic alterations in length.** Three talin molecules are shown in different states, all engaged to an integrin (blue) at the N-terminus. Folded – the 13 rod domains are all in the 0 state. Unfolded – the 13 rod domains are all in the 1 state in the helical form. The positioning of the 11 vinculin binding sites (red helices) is shown. Stretched – the 13 rod domains are all in the fully extended 1 state where it is extended to the completely unstructured polypeptide chain form. The length of the talin molecule ranges from 86 nm (folded) to ~350 nm (unfolded) to 800 nm (stretched). The diameter of a dendritic spine is ~1000 nm. <https://youtu.be/yCOkSaIkAQ4>

**Supplementary Movie 3. MeshCODE – binary information written into the shape of a talin molecule.** A single talin molecule and the corresponding binary string is shown. At start of animation all the switches are in the folded, 0 state. As force is exerted on the molecule, the switches open and close independently and the binary string is updated. N.B. Only the string of helices, 1 state is shown and not the fully extended 1 state, this is for visualisation purposes, in reality each helix in the open 1 state would extend from 5 nm to 12 nm. <https://youtu.be/pqVANarhQi4>

**Supplementary Movie 4. A zone of activity of a protein tethered to the talin R8(0) switch.** The molecule has a tether at one end and an enzymatic domain at the other end. Attached via its tether site, the enzymatic domain can reach anywhere within the sphere but cannot reach outside of that zone. The radius of the sphere is a physical property of the linker region of the enzyme but the location of the tether where that sphere is attached is a function of the talin switch patterns. <https://youtu.be/90WZYos01LU>

**Supplementary Movie 5. A single posttranslational modification (PTM) dramatically alters the MeshCODE dimensions.** A movie showing how a chemical modification to the talin switches can alter the 3-dimensional location of molecules within the cell. A single talin molecule is shown attached to integrin (blue). A zone of influence of a molecule on the R9 switch is shown (grey sphere) positioned 87 nm away from the integrin attachment. The enzyme cyclin dependent kinase 1 (CDK1) is shown interacting with the R8(0) switch, and phosphorylating talin. This phosphorylation destabilises R7R8, and the two domains unfold, introducing a 110 nm extension to the talin molecule. As a result the zone of influence of the molecule on R9 is now 110 nm further from the integrin attachment. <https://youtu.be/LVpwCPePovk>
